# A gamified virtual environment intervention for gait rehabilitation in Parkinson’s Disease: co-creation and feasibility study

**DOI:** 10.1186/s12984-024-01399-6

**Published:** 2024-06-24

**Authors:** Pere Bosch-Barceló, Maria Masbernat-Almenara, Oriol Martínez-Navarro, Carlos Tersa-Miralles, Anni Pakarinen, Helena Fernández-Lago

**Affiliations:** 1 Department of Nursing and Physiotherapy, University of Lleida, Montserrat Roig, 2, Lleida, 25198 Spain; 2 Health Care Research Group (GRECS), Lleida Institute for Biomedical Research Dr. Pifarré Foundation (IRBLleida), Lleida, Spain; 3 Lleida Institute for Biomedical Research Dr Pifarré Foundation, IRBLleida, Lleida, Spain; 4 Consolidated Research Group: Society, Health, Education and Culture Research Group (GESEC), University of Lleida, Lleida, Spain; 5 Department of Nursing Science, University of Turku, Turku, Finland

**Keywords:** Virtual reality, Gamification, Feasibility, Parkinson’s disease, Gait rehabilitation

## Abstract

**Background:**

Treadmill gait training has been shown to improve gait performance in People with Parkinson’s Disease (PwPD), and in combination with Virtual Reality, it can be an effective tool for gait rehabilitation. The addition of gamification elements can create a more stimulating and adherent intervention. However, implementation of new technologies in healthcare can be challenging. This study aimed to develop and evaluate the feasibility of a treadmill rehabilitation program in a Gamified Virtual Reality Environment (GVRE) for PwPD.

**Methods:**

The GVRE was developed following a user-centered design approach, involving both PwPD and physiotherapists in the development and evaluation of the intervention. The intervention consisted of a walking simulation in three different environments (countryside, city, and park), which had a progressive increase in difficulty. To test its feasibility, three sessions were carried out with four PwPD and four physiotherapists. To assess the usability, the System Usability Scale (SUS), Assistive Technology Usability Questionnaire for people with Neurological diseases (NATU Quest) and Simulator Sickness Questionnaire (SSQ) were used. To assess the intervention’s acceptability, feedback and in-game performance was collected from participants.

**Results:**

Results showed the feasibility of the intervention, with a SUS score of 74.82 ± 12.62, and a NATU Quest score of 4.49 ± 0.62, and positive acceptability feedback. Participants showed clear preferences for naturalistic environments, and gamification elements were seen as positive. Difficulty settings worked as intended, but lowered enjoyment of the experience in some cases.

**Conclusions:**

This intervention was successfully shown as a feasible option for the training of gait under Dual Task conditions for PwPD. It offers a safe and replicable environment in which complex situations can be trained. However, further iterations of the intervention need to be improved in order to guarantee accurate tracking and a more realistic training progression.

**Trial registration number:**

NCT05243394–01/20/2022.

## Background

Parkinson’s Disease (PD) is the second most common neurodegenerative disorder in the world, and the fastest growing one, mainly characterized by motor symptoms such as a highly variable, short-stepped gait, as well as resting tremor, bradykinesia, postural instability and rigidity [1, 2]. Non-motor symptoms, particularly cognitive decline, also significantly impact People with Parkinson’s Disease (PwPD) [3]. In situations where a cognitive demand is added to walking, known as Dual Task (DT), PwPD exhibit an increase in gait variability, which points to an increased risk of future falls in PD [4, 5].

The use of antiparkinsonian medications shows improvements on single task walking (i.e., individuals focusing solely on gait) but could limit or hinder cognitive functions that are key for a safe gait under DT conditions [6]. On the other hand, the training of PwPD under DT conditions has shown improvements in gait parameters with no increase on fall risk, and even improving perceived quality of life [7, 8].

New technologies such as Virtual Reality (VR) offer a safe and replicable environment in which to train DT conditions effectively [9, 10]. VR provides an opportunity to incorporate a diverse range of motor, cognitive, sensory, and psychological stimuli (i.e., reacting to changes in the environment, obstacle approach planning and management, adapting to different difficulty levels, distractions, decision-making, etc.). These stimuli can align with the demands of DT scenarios and have demonstrated effectiveness as a gait training tool [11]. The addition of VR environments to well established rehabilitation modalities such as treadmill training [12] could create motivating treatment options that improve adherence for PwPD [13]. Complementing VR with gamification elements can add benefits for elderly people, both in cognitive and physical domains [14]. Nevertheless, there is a notable lack of robust evidence to support the superiority of VR interventions in terms of efficacy and reduced labor intensity compared to non-VR interventions [15]. Furthermore, it is essential to tailor rehabilitation strategies to align with the specific context and foundational skills of the target population to ensure maximum accessibility and benefit [15, 16]. Recent research has pointed out that VR rehabilitation for PD is mostly administered through commercial exergames, such as Nintendo’s Wii Fit and Microsoft’s Kinect Adventures [17, 18]. However, these devices were originally designed for healthy individuals and may not fully address the specific needs of PD patients. PD patients often experience stiffness, slowness, tremors, and cognitive decline, which can impact their ability to interact with commercial videogame features effectively. As a result, the effectiveness of exergame training on mobility and balance performance may be compromised in this population [18, 19]. Moreover, the use of new technologies in neurorehabilitation can present difficulties for patients and healthcare professionals in aspects such as learnability and complexity of the system [20]. Thus, to create a rehabilitation intervention that is both accessible and engaging, it is essential to consider the needs and preferences of end-users. One approach that can help achieve this goal is User-Centered Design, which involves gathering feedback from end-users throughout the development process to improve the system [21, 22].

Aligning User-Centered Design with feasibility testing could be a beneficial method to ensure successful implementation of rehabilitation interventions, as well as a better fit to the real needs of users [23–25]. Moreover, feasibility studies help researchers find whether a complex intervention can be assessed at larger scale, such as with a randomized clinical trial [26, 27].

In this study, we introduce an innovative intervention that utilizes a treadmill rehabilitation program within a custom-designed Gamified Virtual Reality Environment (GVRE) specifically tailored for PwPD. We have sought the insights of both PwPD and physiotherapists as end-users to enhance and fine-tune the intervention. Assessing the feasibility of the developed software and hardware is crucial, particularly in a training context, to ensure adherence to the intervention and its potential effectiveness. Therefore, the main aim of this feasibility study was to develop and evaluate the usability and acceptability of a treadmill rehabilitation program in a GVRE for PwPD. This study was divided into two different phases: one for software design and development and a second one for software implementation and testing within a feasibility study.

## Intervention design and development

The intervention consists of a walking simulation in 3 different environments, which has a progressive increase in difficulty over time based on 5 different parameters: speed, visibility, path width, obstacles, and distractors.

In the first phase, a review of the available literature for technological solutions for the rehabilitation of gait and DT conditions in PwPD was performed.

**Fig. 1:**
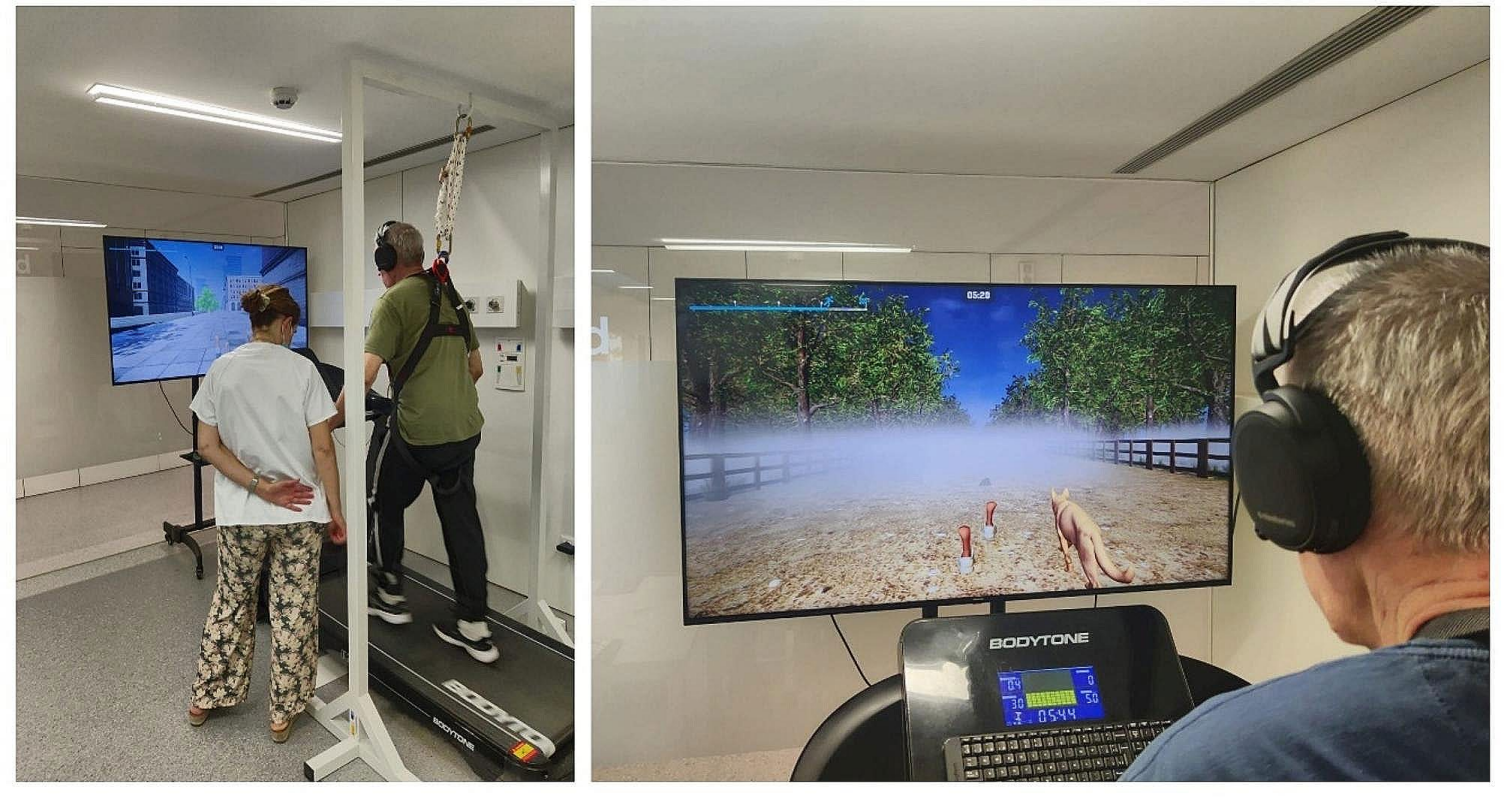
Gamified Virtual Reality Environment + treadmill set-up

**Fig. 2:**
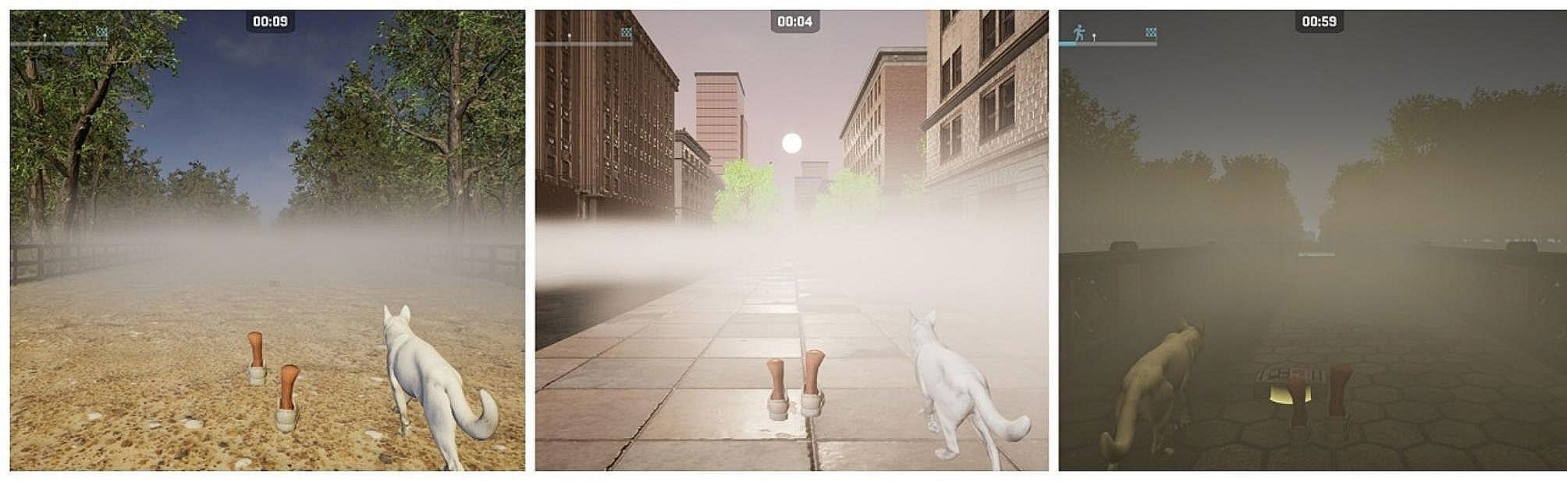
Fog as a visibility limitation to increase difficulty

**Fig. 3:**
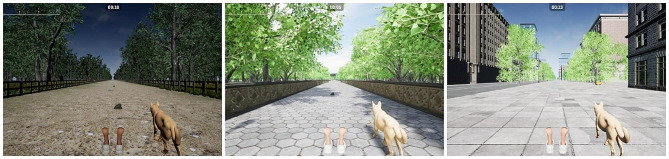
Three different environments: Countryside, Park, City

In the second phase, and by applying Scrum Methodology, the team of physiotherapists, nurses and computing scientists decided to develop an augmented reality treadmill set-up (Fig. 1) based on studies by *Mirelman et al.* [28, 29]. The software was created with Unreal Engine 5 and programmed using C++. During walks, different obstacles such as traffic cones, cardboard boxes or bricks appeared either on the right or left side of the path. Participants had to avoid them by executing a skipping movement with the corresponding foot on the treadmill. As they approached an obstacle, careful planning was necessary to lift the foot corresponding to the obstacle’s side. In later levels, difficulty was increased by the appearance of fog and reduced environmental light to limit visibility (Fig. 2), the use of distractors, and the progressive reduction of the width of the pathway. The progressively narrowing path simulated a cluttered visual field, with trees, buildings, and fences appearing closer to the participant, creating a denser environment. These challenging scenarios may exacerbate gait disturbances and provoke freezing episodes in PwPD. The application of treadmill training, enhanced by auditory and visual stimuli, has been explored as a rehabilitative strategy [30]. The validation of the following elements composing the software was performed during this phase: Three different environments (Countryside, City and Park) (Fig. 3) with their environmental sounds, an initial User-Interface, a performance-based level progression, different obstacle designs, distractors and modifiers of visibility. The specific distractors used were based on each environment as to provide a more coherent experience: in the countryside environment, participants could be distracted by foxes and other animals crossing their path or standing and moving on the sideway; on the city environment, cars ran along the side of the road and construction noises could be heard; in the park environment, footballs could cross the walking path, and birds could take flight in front of the participant as they approached them. The appearance of distractors was set on specific windows of time for each bout. The simulation moved forward on a straight line at the predetermined speed set to each training session independently of the participant’s movements, and would keep moving until paused or stopped.

In the third phase, technical development and refining of the software was performed. An iterative, User-Centered Design process [31] was followed in this phase, by gathering feedback from two different physiotherapists with expertise in PD and neurorehabilitation and by one PwPD. By testing the set-up via Human-Computer Interaction, the tracking system was validated and switched from whole-body cameras to HTC Vive technology (HTC Trackers 3.0 & HTC SteamVR sensors) to improve accuracy on participants’ movement. The User Interface was also validated to allow changes to obstacle frequency, visibility limitations, tracker calibration, speed customization, level selection and data management. Setting up the tracking system, calibrating it, selecting the session and parameters on the interface and securing a participant with the safety harness could be done in approximately 5 min.

Gamification elements to promote adherence and motivation for participants were also incorporated in this phase. In the game, participants performed a “walking my dog” task, during which they walked along the different environments and experienced the surroundings, which included scoring (by successfully avoiding obstacles), feedback messages, visual hints, progress bars (showing the progression along the walking bout and the distance to the finishing line), customization, goal setting and adjustable difficulty, was incorporated to promote participants’ motivation and adherence. The dog walked on a straight line besides the participant, and could be on the right or left side of the participant, based on preference. There was also the chance to remove the dog and walk alone for participants. The inclusion of gamification elements in the GVRE software is justified by the Self-Determination Theory proposed by Ryan and Deci [32]. This theory emphasizes the role of autonomy, competence, and relatedness in fostering intrinsic motivation [33] (Table 1).

**Table 1:** Challenges, gamification heuristics, and game mechanics to address them

Challenge	Heuristic	Game mechanics
Support learner’s autonomy	Provide a moderate amount of meaningful option	When first logging their profile into the software, participants are able to name the dog and assign it a color from four different color pallets.
Support learner’s competence	Set challenging but manageable goals	Level of difficulty in the virtual reality environment can be modified through 5 different mechanisms: speed (as explained previously), obstacles, distractors, visibility, and path width.
	Provide positive, competence-related feedback	Participants can go up one level after the first session if they complete 3 bouts with over 80% success in obstacle management, with certain limitations. Obstacles show a yellow circle surrounding them when participants are getting close to them to indicate participants when to dodge, changing color to green if they are skipped over successfully, and to red if they are hit (Fig. 4).
Support learner’s relatedness	Facilitate social interaction	The “walking my dog” task and the end-of-level messages create a sense of relatedness by taking care of the pet and setting a narrative around the dog needing rest after a good walk.
Individual characteristics	Make the system flexible	The system adapts to the characteristics of each participant by using their performance as the main driver of progression within the levels.

**Fig. 4:**
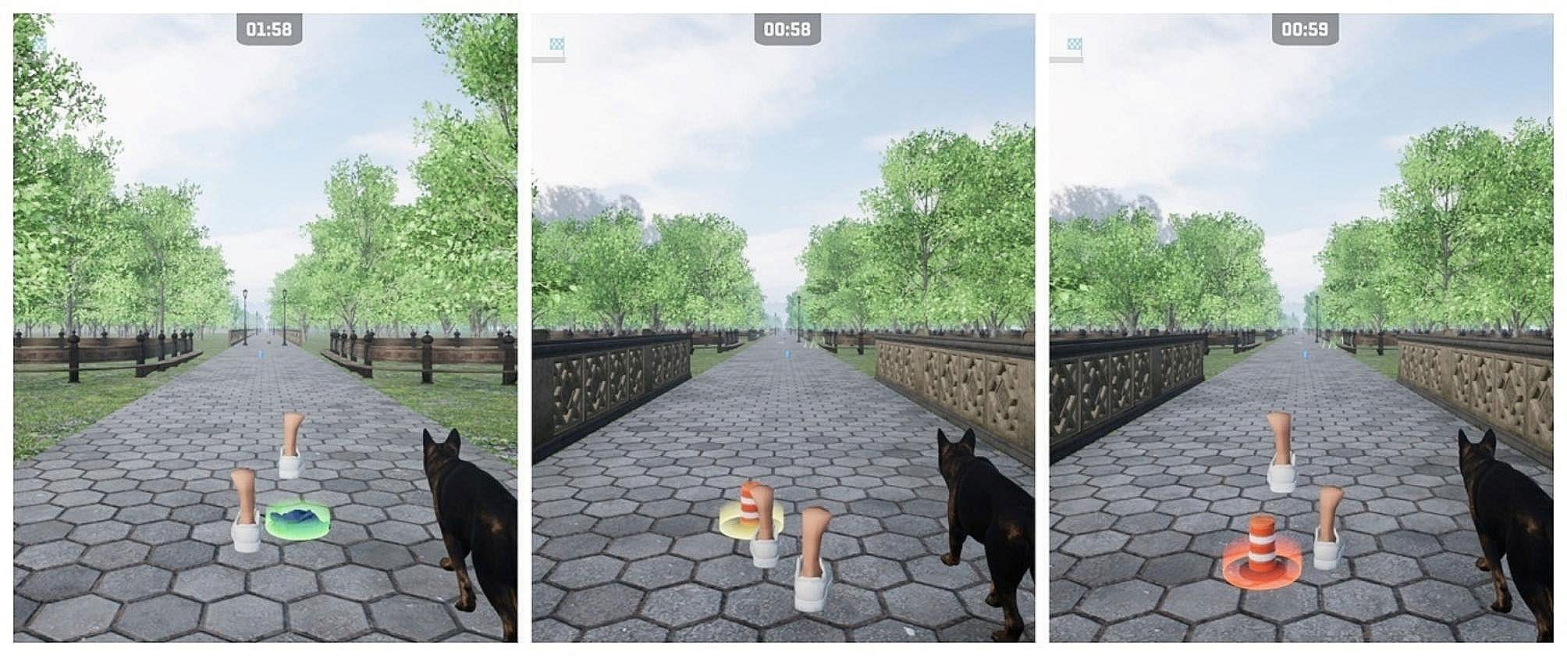
Obstacle dodging (green – correct; yellow – approaching; red – collision)

## Feasibility study

A single arm, one group, pre-post feasibility study was conducted. All interventions and data collection were performed at Biomedical Research Institute of Lleida (IRBLleida). The study began in May 2022 and lasted until June 2022.

### Participants

A total of eight participants were recruited, comprising four participants with PD and four physiotherapists. Participants with idiopathic PD (based on UK Parkinson’s Disease Society Brain Bank Diagnostic Criteria [34] were recruited from the local Parkinson Disease association through convenience sampling. Inclusion criteria for this group of participants were: (1) age between 45 and 80; (2) Stage II to III in the Hoehn&Yahr scale (H&Y); (3) ability to walk for 10 or more minutes unassisted; (4) MiniMental State Examination (MMSE) score over 24 points. Exclusion criteria included significant cognitive decline based on MMSE (< 23), severe auditory or visual deficits, other neurological or psychiatric conditions, any kind of cardiovascular complications that contraindicates physical activity and a clinical history of any brain surgery or use of a deep brain stimulation device. Participants were tested and trained on their regular dopaminergic medication. To avoid circadian effects, the training and testing sessions including the post-training assessment took place at the same time of the day in every session.

Physiotherapists with different experience and expertise (a private practice physiotherapist, two physiotherapists specialized in neurology, and a geriatrics physiotherapist) working with neurological conditions or specifically PD were also recruited to supervise the sessions and test the system from their point of view.

All participants gave their signed informed consent to participate in the study. All procedures had been approved by the ethical committee of Hospital Universitari Arnau de Vilanova (CEIC-2231), and both the Helsinki Declaration and the Oviedo Convention were followed [35, 36]. Spanish regulations regarding Biomedical Research were met as well [37].

### Intervention implementation

All participants participated in three different sessions, where all available content for the GVRE was tested and with layering of the different difficulty settings. Participants started with a small 2-minute warm-up on the treadmill with no VR at the beginning of every session. Sessions were scheduled once a week. Walking speed increased based on preferred ground walking speed as levels progressed. In situations where participants had no previous experience with treadmill gait training, a familiarization period was performed without VR.

To determine the participants’ preferred ground walking speed, a 10-meter walking test on the ground was performed, and speed calculations were used to determine the speed for the levels required: 90% of preferred speed for session 1, 110% for session 2, and 130% for session 3. The walk duration was 4 bouts of 5 min for a total of 20 min in the 1st session, 3 bouts of 7 min for a total of 21 min in the 2nd session and 2 bouts of 9 min for a total of 18 min in the 3rd session. 2-minute period rests were included between bouts. To increase the training difficulty as sessions progressed, we adjusted obstacle frequency and path width. Specifically, obstacles appeared every 30 s, and the path width was 4 m in session 1. In session 2, obstacles appeared every 25 s, and the path width reduced to 3 m. Finally, in session 3, obstacles appeared every 20 s, and the path width reduced to 2 m. Distractors appeared on bouts 2, 3 and 4 in session 1, bouts 2 and 3 in session 2 and bout 2 in session 3. Additionally, we introduced fog and reduced environmental light as the sessions progressed. Bouts 2 and 3 in session 2 had a moderate limitation in visibility, while bout 2 in session 3 had a more severe visibility limitation. This training set-up was chosen to test the widest possible array of parameters while keeping sessions brief and avoiding longer bouts, since participants were not used to the training duration as much as participants in longer studies would be.

### Data collection and analysis

To test the usability and acceptability of the intervention, quantitative assessments and qualitative observations of participants’ feedback were integrated. This mixed methods approach has already been successfully used in testing the usability and acceptability of other exergames [38].

#### Demographic and clinical descriptors

PwPD completed the Montreal Cognitive Assessment (MoCA) as well as the United Parkinson’s Disease Rating Scale (UPDRS) and the Balance Evaluation Systems Test (MiniBEST). Information on physical activity was collected through the International Physical Activity Questionnaire (IPAQ) and overall disease status through H&Y scale. All clinical assessments were performed before the beginning of the first session.

#### Usability measures

Physiotherapists and PwPD completed the following measures to learn about the intervention’s usability:

- **System Usability Scale (SUS)** [39] is a 10-item questionnaire that provides a quick, reliable tool for measuring usability on a score from 0 to 100.
- **Assistive Technology Usability Questionnaire for people with Neurological diseases (NATU Quest)** [40] is a measure scale designed to assess the usability of assistive technology in people with neurological diseases on a score from 0 to 5.
- **Simulator Sickness Questionnaire (SSQ)** [41] is an assessment tool used to measure cybersickness or adverse symptomatology related to VR usage. In our augmented reality and treadmill setting, several sickness symptoms such as eye fatigue, disorientation and nausea can appear due to hardware, virtual content or human factors [42]. The SSQ provides specific scores regarding Nausea, Oculomotor disturbance and Disorientation. The sum of all sub scores provides the total score.
- **Independent Television Commission Sense of Presence Inventory (ITC-SOPI)** [43] is a 44-item questionnaire that measures the experience of users in certain displayed environments using a 5-point Likert scale. Since the ITC-SOPI does not have a validated version in Spanish or Catalan, it was translated to Spanish by the team and reviewed by a professional translator.

SSQ was the only measure assessed before and after each intervention. The SUS and the ITC-SOPI were filled after each session, and the NATU Quest was assessed after finishing the final session. Physiotherapists also filled the SUS and the ITC-SOPI after each session. Filling these two different measures every session allowed for detailed information on each of the city, countryside and park environments.

#### Acceptability measures

The acceptability of the intervention was assessed by measuring the performance of participants within the GVRE. This was indicated by their ability to dodge obstacles successfully. This performance metric worked as an additional indicator of the intervention’s acceptability, as a reflection of how the different elements within the simulation, such as difficulty settings, interact with the ability of PwPD to manage obstacles.

Moreover, both physiotherapists and PwPD gave their constant feedback throughout sessions, in which the “Thinking Aloud” technique was applied, as well as answering to open-ended questions by the researchers.

#### Data analysis

Quantitative analysis of the different questionnaires filled was calculated by using measures of dispersion, mainly mean and standard deviation values, by using the GNU PSPP software. Qualitative analysis of the information gathered via feedback by participants in the acceptability field was analyzed via inductive content analysis. All relevant information mentioned during conversation within each session was codified, then grouped into categories by theme.

## Results of the feasibility study

### Demographic and clinical descriptors

4 participants with Parkinson’s disease were involved, with a mean age of 61 ± 9 years. Demographic data for all participants is summarized in Table 2.

**Table 2:** Demographics

Participant Group	PwPD	Physiotherapists
Number of Participants	4	4
Gender Distribution	1 Female, 3 Male	2 Female, 2 Male
Mean Age (years)	61 ± 9	37.5 ± 7
Computer Experience	Basic to Intermediate	Basic to Expert
Virtual Reality Knowledge	Intermediate to None	Basic to None
H&Y Disease Stage	2 at Stage 2, 2 at Stage 3	-
MoCA Score	27 ± 2.5	-
UPDRS Score	54.5 ± 22.5	-
MiniBEST Score	21 ± 5.2	-
Physical Activity Level (IPAQ)	1 Vigorous, 1 Low, 2 Moderate	-

### Usability

The average usability score for the software based on the SUS was good (74.82 ± 12.62). PwPD gave a higher score than physiotherapists (77.36 ± 8.62 vs. 72.49 ± 13.86). The highest usability scores were given on Countryside environment sessions (76.83 ± 10.21), and the lowest ones were given on Park environment sessions (72.63 ± 13.8).

The usability score for the set-up given by PwPD according to the NATU Quest was 4.49 ± 0.62. Scores for each individual question can be found in Table 3.

**Table 3:** NATU quest scoring

Question	Mean	STD
1. I believe that this rehabilitation tool can help me improve my functional independence.	5,00	0,00
2. I feel comfortable using this rehabilitation tool.	4,71	0,43
3. This rehabilitation tool adapts to my characteristics and needs.	4,71	0,43
4. Donning this rehabilitation tool is quick and easy for me.	4,71	0,43
5. I feel safe using this rehabilitation tool.	4,29	0,87
6. This rehabilitation tool allows me to achieve my goal/ allows me to perform a movement/action I could not do before.	4,44	0,50
7. This rehabilitation tool adapts to my special needs.	4,07	0,83
8. In general, this rehabilitation tool is easy to use.	4,29	0,87
9. Information and instructions of use of this rehabilitation tool are easy to understand and easy to remember.	4,07	0,83
10. Overall, I am satisfied with this rehabilitation tool.	4,71	0,43

The SSQ reported a score of 14.03 ± 7.99, which falls into the “significant level of sickness” after the intervention. However, the total score was mainly increased due to higher values on the Fatigue and Sweating subcategories, both expected while performing rehabilitation on a treadmill. The scoring for the categories of the SSQ were as follows: Disorientation: 1.79 ± 5.02; Oculomotor: 13.27 ± 6.83; Nausea 25.23 ± 18.56.

The ITC-SOPI yielded total scores of 2.33 ± 0.63 for Spatial Presence, 2.87 ± 0.50 for Engagement, 3.20 ± 0.74 for Ecological Validity/Naturalness, and 0.58 ± 0.23 for Negative Effects. Detailed information on the scores for each participant subgroup and specific environments can be found in Table 4.

**Table 4:** ITC-SOPI subgroup and environment scoring

Spatial presence	Mean	STD
PwPD	2,43	0,79
Physiotherapists	2,24	0,41
Countryside Environment	2,34	0,63
City Environment	2,55	0,62
Park Environment	2,18	0,43
**Engagement**		
PwPD	2,65	0,48
Physiotherapists	3,09	0,51
Countryside Environment	2,67	1
City Environment	3,02	0,83
Park Environment	2,91	0,79
**Ecological Validity/Naturalness**		
PwPD	3,25	0,97
Physiotherapists	3,15	0,38
Countryside Environment	3,23	0,6
City Environment	3,48	0,37
Park Environment	3,08	0,23
**Negative Effects**		
PwPD	0,85	0,26
Physiotherapists	0,31	0,19
Countryside Environment	0,63	0,71
City Environment	0,58	0,83
Park Environment	0,48	0,8

### Acceptability

#### Dodging obstacle performance

The performance of PwPD was evaluated based on the participants’ ability to successfully dodge obstacles encountered during the walking bouts. The number and frequency of obstacles increased progressively over the course of the study, with a total of 9 obstacles on each walking bout of Day 1, 16 on each bout of Day 2, and 27 on each bout of Day 3. Participants showed a tendency to improve their dodging performance as bouts progressed during Day 1. For Day 2 and Day 3, a reduction in performance appeared the moment the visibility limitation bouts were introduced. Detailed information on each bout and obstacle dodging can be found in Table 5.

**Table 5:** Bout information and percentage of successfully dodged obstacles

	Duration	Obs. Fx	Distractors	Visibility	P1	P2	P3	P4
**Day 1 – City**								
B1	5’	30”	Cars and construction noise	No limitation	100%	66,67%	33,33%	88,89%
B2	5’	30”	Cars and construction noise	No limitation	88,89%	77,78%	44,44%	88,89%
B3	5’	30”	Cars and construction noise	No limitation	100%	77,78%	55,56%	77,78%
B4	5’	30”	Cars and construction noise	No limitation	88,89%	100%	N/A	100%
**Day 2 – Countryside**								
B1	7’	25”	Animals on side; foxes crossing path	No limitation	57,14%	87,50%	81,25%	93,75%
B2	7’	25”	Animals on side; foxes crossing path	Moderate limitation	93,75%	62,50%	75%	81,25%
B3	7’	25”	Animals on side; foxes crossing path	Moderate limitation	81,25%	62,50%	56,25%	75%
**Day 3 – Park**								
B1	9’	20”	Birds and footballs	No limitation	81,48%	65,38%	76,92%	57,69%
B2	9’	20”	Birds and footballs	Severe limitation	85,18%	46,15%	53,85%	43,75%

*B: Bout; Obs. Fx: Obstacle Frequency; P: Participant; ‘: minutes; “: seconds;*

#### Qualitative data

Five categories for intervention acceptability were identified after qualitative analysis.

#### Hardware and safety

Participants expressed discomfort and distraction from the harness system that restrained them while walking on the treadmill. However, it was also perceived as a comforting piece of the set-up, reassuring them as they walked. They also expressed a preference for leaning on the treadmill or holding on with their hands to feel more secure. Physiotherapists suggested improvements to the safety system, such as the use of parallel bars for better support.

PwPD1: “It [the harness] bothers me, I can’t walk normally with it”.

PwPD4: “I feel safe with the harness and leaning on the treadmill”.

#### Virtual environments and gamification

Participants showed a preference for the Countryside environment over the City environment, as they considered it more realistic and pleasant. They also appreciated the presence of animals and sounds in the environment, although ravens as distractors were perceived as creepy or disturbing. The dog was perceived as a reassuring and calming experience, and its speed and distance from participants may be used as progress feedback as training advances. Physiotherapists suggested increasing the realism of the scenarios with avatars of people walking by, changes of pavement and surface, and a wider variety of obstacles. They also suggested different gamification options such as customizing the shoes and the dog and introducing data such as distance walked, or steps taken. The use of a curved TV to improve the immersion experience was also suggested.

PwPD2: “The noise of the construction site was distracting. (…) I felt calm about the cars, they didn’t cross my way”.

#### Motor planning

Participants reported difficulty in negotiating obstacles due to the constant speed of the treadmill, the lack of visibility due to fog, and the size of the obstacles. They also expressed a desire for better accuracy when avoiding obstacles by introducing changes in the shape of the hitbox, allowing for a more realistic approach. Physiotherapists indicated that obstacle avoidance forced the movement of the most affected lower limb, which was positive for rehabilitation. In addition, they proposed randomizing the sequence in which the obstacles appeared and adjusting the height of the obstacles to adapt the difficulty level.

PT3: “Having them [the obstacles] appear at random instead of always in order would be nice”.

#### Training variables

Participants experienced a feeling of fast speed on the treadmill until they got used to it, and some felt that the preferred speed was too high for real life. They also reported tiredness and fatigue during breaks, and that walking on the treadmill was more tiring than on the floor. Physiotherapists proposed to automatically calculate the speed within the participant’s profile and to automate its progression based on performance.

PwPD2: “I can do it [walk at the required speed] but I don’t need to go this fast on the street”.

PwPD1: “I had to be so attentive to the step length and the speed, that the obstacles… I didn’t have time to recalculate towards them”.

#### Improvements for physiotherapists

Physiotherapists suggested saving the information of tracker calibration within participants’ profiles, as well as knowing the percentage error rate of each foot when managing obstacles. They also proposed counting the distance walked and steps taken by participants, as well as making the time numbers in each block bigger and the progress bar more visible. They indicated the desirability of increasing the variety of obstacles, environments, distractors and surfaces to regulate the difficulty and progression of the training.

PT3: “You could have a “save” option for the tracker configuration, so you don’t have to calibrate them each time”.

## Discussion

The aim of this study was to evaluate the usability and acceptability of the intervention from the perspective of PwPD and physiotherapists. This intervention was successfully shown as a feasible option for gait rehabilitation under complex conditions for PwPD. This involved an interplay between a motor task, exemplified by treadmill walking, and cognitive planning of various stimuli, including obstacle avoidance and the management of visibility constraints and distractors, aligning with the principles of DT training [11]. It posed a safe and replicable environment in which complex situations could be trained. Further iterations of the intervention need to be improved to guarantee accurate tracking and a more realistic training progression. This study could point other researchers and developers to the creation of more accurate solutions for the rehabilitation of PwPD.

The decision to use HTC SteamVR sensors linked to two HTC Vive 3.0 Trackers was taken to gain more accuracy on the movement of the participants’ feet. This technology is relatively recent, but widely used in health interventions [44]. More precise tracking means a better experience in interacting with obstacles, which is a key part of the proposed GVRE. Overall experience was positive, and walking and obstacle dodging was fluid and precise most of the time. However, the tracking system had issues in some situations, mainly with intermittent losses of information, which caused the virtual feet to freeze. In these situations, basic interaction with the GVRE could not be performed, and the participant experiences a disconnection from the immersive experience. With such key mechanics within the game not working accordingly 100% of the time, methodical analysis of the software and hardware interaction is needed to fix this sort of issue.

The results of this study showed that the described intervention is a usable option for both Physiotherapists and PWPD. This result is represented by a 4.49/5 score on the NATU Quest pointing to a usable set-up, and a “Good” ranking for the software on the SUS. Usability scores remained high even for inexperienced participants in treadmill training and computer and VR knowledge, and the intervention proved viable for different stages of progression of PD within the H&Y scale. This result is consistent with previous research on Virtual Reality, pointing to an effective therapy that can be motivating, fun and engaging for PWPD [16].

Assessment of cybersickness through the SSQ reported a significant level of sickness after the intervention. However, it should be noted that most of the scores rising the sickness level correspond to Fatigue and Sweating on the categories of Oculomotor and Nausea, both expected on a physically demanding activity such as walking on a treadmill while engaging in a virtual environment. Furthermore, prior research [45] has suggested that the symptom thresholds established by the SSQ were initially established based on a demographic that is not representative (military aviation pilots), potentially biasing the general population towards higher scores. Previous studies have reached similar conclusions regarding this topic [46]. Taking this into account, this intervention is considered to be safe and not prone to provoking cybersickness, despite the “significant level of symptoms” score obtained.

The experience of VR immersion within the GVRE was measured through the ITC-SOPI. Scores were similar between physiotherapists and PWPD, with Ecological Validity reaching the highest scores, while Spatial presence and Engagement scored slightly lower, all compared to mean scores for media samples on the ITC-SOPI testing for Computer Games [43]. Negative effects scores were very low for both groups, but specially so for Physiotherapists. Scores were also similar for all the three different environments in which sessions were performed in all four categories. Lower scoring on Spatial Presence may be related to the use of standard display methods (i.e. a 65” Screen) instead of a Virtual Head-Mounted Display, which has been shown to provide more immersive experiences [47].

Participants demanded a more interactive, realistic and immersive experience. Comments about being able to interact more with the surroundings, like the dog or the elements passed during the walking bout. Participants clearly commented on their preference of Countryside and Park environments over the city environment, with comments about the surroundings being more lively and real. Recent research has pointed to nature environments in VR as a potential tool for mood improvement and stress reduction, which could be linked to the preference of participants for more naturalistic settings [48].

There was also a demand for more gamification elements. The addition of gamification elements to gait training is a powerful resource that can turn a generally boring task into an enjoyable one [49]. As such, careful thought must go onto the inclusion of future elements, which should lead to an enjoyable and motivating experience in future software updates.

Other gamification elements that were seen as beneficial was the inclusion of the dog avatar. The dog was reassuring for participants while having it on their most affected side and provided calmness to the experience. Future software iterations could use the dog animation as a progress indicator, by changing its animation from walking to jogging to running as participants advance through sessions.

The preset speed increases were perceived as being too high. In some cases, corresponding increases in speed could not be applied due to excessive fall risk. The justification for the overestimation of speed probably stems from the fact that the original protocol on which the authors based this study was designed for adults at high risk of falling, who experienced 2 or more falls in the previous 6 months to the study [29]. The population recruited for this study may not be comparable to these mentioned conditions, thus having less margin for improvement compared to the sample from the mentioned study. As such, expectations of speed increase need to be moderated for future study iterations, aiming at lower increases of speed for each week of training.

The performance results in the GVRE met the anticipated expectations. Participants generally showed an improvement in their obstacle management along the first session as they became more familiar with the environment and movement patterns. In the second and third sessions, it became clear that the appearance of fog as a reduction in visibility and reduction in path width, which were aimed at increasing difficulty, successfully affected the performance of the participants, causing an increase in obstacle collisions. This expected reduction in performance confirms that the difficulty settings worked as intended. Comments about how fog affected their planification while dodging confirmed this hypothesis. However, the limitation in visibility also affected their enjoyment of the environments, with thicker fog having participants enjoy the settings less. In this context, the difficulty settings worked correctly, but had clear drawbacks that should be considered. Nevertheless, in a prolonged training scenario where participants are granted an extended duration of time to learn and navigate the complexities of the environment, it is anticipated, in accordance with the Self-Determination Theory [32], that they would be motivated to strive for improved outcomes and be compelled to exceed prior scores despite the escalating difficulty. Future research should study how the introduction of difficulty elements affect PwPD and whether they have an impact on motivation and adherence.

## Strengths and limitations

This study is one of the first to develop and test a customized GVRE for treadmill gait training in PwPD. The study followed a user-centered design approach, involving both PwPD and physiotherapists in the development and evaluation of the intervention. A mixed methods approach was used, combining quantitative and qualitative data to assess the usability and acceptability of the intervention. Moreover, the intervention relied on widely available and affordable hardware components, which facilitates replication of the intervention.

However, this study had some limitations that should be acknowledged. The intervention was tested only for three sessions, which does not allow PwPD to achieve training adaptations as they would in an intervention with a larger training volume. This could bias the feedback obtained regarding training periodization, rest periods, and overall fatigue and effort. There was no control over any other physical activities performed by participants throughout the rest of the day, which may have conditioned performance in some cases. Since the ITC-SOPI does not currently have a validated version in Spanish, a translation by the authors was performed and subsequently checked by a professional translator. However, biases could be derived from imperfect translations and results from this questionnaire should be interpreted with caution. DT stimuli in the simulation were mainly based on cognitive and motor planning towards obstacle management and reduced reaction time through visibility impairments and distractors; however, no decision-making inputs or direction changes were introduced, which would provide a more enriching experience for participants.

## Conclusions

In conclusion, this study provides an insight into the design, development, and feasibility of a rehabilitation intervention for PwPD based on treadmill training and VR. This intervention proved feasible both for PwPD and physiotherapists. This set-up is a strong option to help PwPD better manage their difficulties in DT conditions in real life by training complex situations in a safe and replicable environment. However, improvements based on the feedback gained from this study need to be considered and further evaluated: tracking in this context needs to be as precise as possible, since it is the main point of interaction for participants, and training periodization needs to be adjusted and individualized to the physical condition of each subject.

## Data Availability

All data generated or analyzed during this study will be made available upon reasonable request.

## Declarations

Participants gave their individual signed informed consent to participate in the study and were thoroughly informed about all procedures by a researcher.

## Abbreviations

PD: Parkinson’s Disease
DT: Dual Task
VR: Virtual Reality
PwPD: People with Parkinson’s Disease
GVRE: Gamified Virtual Reality Environment
MMSE: MiniMental State Examination
H&Y: Hoehn and Yahr Scale
MoCA: Montreal Cognitive Assessment
UPDRS: United Parkinson’s Disease Rating Scale
MiniBEST: Balance Evaluations Systems Test
IPAQ: International Physical Activity Questionnaire
ITC-SOPI: Independent Television Comission – Sense of Presence Inventory
SUS: System Usability Scale
NATU Quest: Assistive Technology Usability Questionnaire for people with Neurological diseases
SSQ: Simulator Sickness Questionnaire
